# *ZFAS1*: a novel tumor-related long non-coding RNA

**DOI:** 10.1186/s12935-018-0623-y

**Published:** 2018-09-03

**Authors:** Dan Dong, Zhongyi Mu, Chenghai Zhao, Mingli Sun

**Affiliations:** 1 Department of Pathophysiology, College of Basic Medical Science, China Medical University, Shenyang, People’s Republic of China; 2 Department of Pharmacology, School of Pharmacy, China Medical University, Shenyang, People’s Republic of China; 3 Department of Urology, Liaoning Cancer Hospital & Institute, Cancer Hospital of China Medical University, Shenyang, People’s Republic of China

**Keywords:** Long non-coding RNA, *ZFAS1*, Diagnostic biomarker, Therapeutic target

## Abstract

Long non-coding RNAs (lncRNA) are classified as a kind of RNA, which are longer than 200 nucleotides in length and cannot be translated into proteins. Multiple studies have demonstrated that lncRNAs are involved in various cellular processes, including proliferation, differentiation, cell death, and metastasis. In addition, aberrant expression of lncRNAs has been discovered in human tumors, where they function as either oncogenes or tumor suppressor genes. Among numerous lncRNAs, we focus on ZNFX1 antisense RNA 1 (*ZFAS1*), a well-known lncRNA that is aberrant overexpression in various tumors, including melanoma, esophageal squamous cell carcinoma, non-small cell lung cancer, gastric cancer, colon cancer, and Hepatocellular carcinoma, in which it functions as oncogene. In contrast, *ZFAS1* is downregulated in breast cancer, which may function as tumor suppressor gene. In this review, we provide an overview of current evidence concerning the role and potential clinical utilities of *ZFAS1* in human cancers.

## Background

Cancer incidence and mortality are increasing each year, making cancer a major public health problem and the leading cause of death worldwide. It is reported that 4,292,000 new cancer cases and 2,814,000 cancer deaths are predicted to occur in China in 2015 [1] and 1,688,780 new cancer cases and 600,920 cancer deaths will occur in United States in 2017 [2]. Despite current advances in surgery, chemotherapy, radiotherapy, targeted therapy and immunotherapy, cancer remains a leading cause of death and constitutes an enormous burden worldwide [3, 4]. One of the reasons affecting the tumor prognosis was lacking of effective early diagnostic method before progression and effective prognostic indicator for guiding the clinical treatment. Therefore, identifying potential diagnostic and prognostic biomarkers for cancer patients to guide clinical decision is crucial and necessary.

Long noncoding RNAs (LncRNAs) are a class of non-protein coding RNA molecules with more than 200 nucleotides in length, which were considered as genomic “junk” and “noise” initially. Thanks to recent advances in sequencing technologies and large-scale genome sequencing projects, more and more lncRNAs have been recently identified in the genomes [5–8]. According to GENCODE analysis (http://www.gencodegenes.org) of the last version of the Ensembl human genome annotation (GRch38, version 24 from August 2015), 28,031 transcripts originating from 15,941 genes can be identified as lncRNAs [9]. Increasing evidences have demonstrated that lncRNAs actively participate in the development of human diseases through regulating the expression of their downstream targets [10, 11]. In tumorigenesis, lncRNAs could function as oncogenes or tumour suppressor genes [12, 13]. Pertinent to clinical practice, lncRNAs have been reported to serve as diagnostic or prognostic biomarkers or as therapeutic targets in many types of human cancers [14, 15]. ZNFX1 antisense RNA 1 (*ZFAS1*), an lncRNA originally identified a regulator of mammary development [16]. Subsequent studies also demonstrated that *ZFAS1* expression is upregulated in many human malignancies, including glioma, lung, colon, liver, ovary and gastric cancers [17], but downregulated in breast cancer [16, 18, 19]. The present review summarizes the current studies of regulation mechanisms and functions of *ZFAS1* in the initiation and progression of human cancers (Tables 1, 2).

**Table 1:** Functional characterization of ZFAS1 in various tumors

Tumor type	Expression	Functional role	Related gene	Role	References
Breast cancer	Downregulation	–	–	Tumor suppressor	[16, 19, 21, 22]
Glioma	Upregulation	Proliferation, migration invasion, cell cycle, EMT process	E-Cadherin, N-cadherin, Snail, MMP2, MMP9, Integrin β1, ZEB1, Twist, HES-1, NICD	Oncogene	[25, 26]
Colorectal cancer	Upregulation	Proliferation, cell cycle, invasion, apoptosis, EMT process	miR-484, p53, cyclin B1, PARP, ZEB1, E-cadherin, ZO-1, Vimentin, N-cadherin	Oncogene	[30–33]
Gastric cancer	Upregulation	Proliferation, cell cycle, migration, apoptosis, EMT process	N-Cadherin, Vimentin, ZEB1, Snail, MMP14, Twist, E-cadherin, EpCAM, Cyclin D1, Bcl2, Bax, Slug,	Oncogene	[37–39]
Hepatocellular carcinoma	Upregulation/downregulation	Proliferation, apoptosis, invasion, metastasis	ZEB1, MMP-14, MMP16, miR-9, miR-150	Oncogene/tumor suppressor	[41, 42]
Ovarian cancer	Upregulation	Proliferation, migration, chemoresistance	miR-150, Sp1	Oncogene	[45, 46]
Melanoma	Upregulation	Proliferation	Ki67, PCNA, CyclinD1	Oncogene	[47]
Non-small cell lung cancer	Upregulation	–	–	Oncogene	[48]
Osteosarcoma	Upregulation	Proliferation, invasion	miR-200b, miR-200c, BMI1, Sp1, ZEB2	Oncogene	[49]
Esophageal squamous cell carcinoma	Upregulation	–	–	Oncogene	[50]
Acute myeloid leukemia	Upregulation	Proliferation, apoptosis	–	Oncogene	[51]
Natural killer/T-cell lymphoma	Upregulation	Proliferation apoptosis, cell cycle	NF-kB, P53	Oncogene	[52]

*ZFAS1* ZNFX1 antisense RNA 1, *EMT* epithelial–mesenchymal transition

**Table 2:** Clinical significance of ZFAS1 in various tumors

Cancer type	Clinicopathological features	References
Glioma	Advanced tumor stage and poor prognosis	[25, 26]
Colorectal cancer	Lymphatic invasion, advanced TNM stage, vascular invasion, poor prognosis	[31–33]
Gastric cancer	Lymphatic invasion, advanced TNM stage, vascular invasion, poor prognosis	[38, 39]
Hepatocellular carcinoma	Intrahepatic invasion, extrahepatic invasion, poor prognosis	[41]
Ovarian cancer	Insensitivity of platinum-based chemotherapy	[45]
Melanoma	Tumor thickness, lymph node metastasis, advanced tumor stages, poor prognosis	[47]
Non-small cell lung cancer	Advanced tumor differentiation grade, lymph node metastasis, advanced TMN stage, poorer prognosis	[48]
Osteosarcoma	Poor prognosis	[49]
Esophageal squamous cell carcinoma	Advanced histological grade, advanced T stage, poor prognosis	[50]

*ZFAS1*: ZNFX1 antisense RNA 1, *TNM* tumor-node-metastasis

## Discovery of *ZFAS1*

*ZFAS1*, located at chromosomal band 20q13.13, an lncRNA that transcripts from the antisense strand near the 5′-end of the protein-coding gene Znfx1, hosts three C/D box snoRNAs (SNORDs): Snord12, Snord12b, and Snord12c [16]. *ZFAS1* was originally identified a regulator of alveolar development and epithelial cell differentiation in the mammary gland [16], subsequently studies showed *ZFAS1* was aberrantly down-regulated in breast cancer tissues and cells [16, 18, 19]. Recent studies have demonstrated that *ZFAS1* was overexpressed in various cancer tissues and cell lines, and promoted the cancer progression through affecting the phenotypes of cancer cells and molecular pathways.

## *ZFAS1* in various cancers

### Breast cancer

Breast cancer is the most frequent malignancies in women worldwide in 2017 based on the latest report on American Cancer Society [2]. Although the improvement in early diagnosis and more effective therapeutic strategies have enhanced the survival rates in past decade, the tumor progression of recurrence and metastasis is still unavoidable [20], and the underlying mechanisms are still unclear. Therefore, it is imperative to explore and elucidate the underlying mechanism and identify new biomarkers for treatment of breast cancer.

The expression and function of *ZFAS1* in breast cancer is different from that in other cancers. Askarian-Amiri et al. [16] reported *ZFAS1* was overexpressed in the mammary gland and down-regulated in breast tumors. Zhang et al. [19] found *ZFAS1* was negative or weakly expressed in breast cancer. Lee et al. [21] found *ZFAS1* was downexpression in HER2-positive breast cancer cells. Fan et al. [22] revealed that *ZFAS1* expression was significantly downregulated in breast cancer cell lines and *ZFAS1* overexpression significantly suppressed cell proliferation, migration and invasion. These findings indicated that the *ZFAS1* may be a tumor suppressor in breast cancer, and thus, may serve as a potential therapeutic target for patients with breast cancer. There is no any reports about the reason for its different role in breast cancer from other cancers until now. According to the present studies that *ZFAS1* was a regulator of alveolar development and epithelial cell differentiation in the mammary gland, we speculated that downregulated *ZFAS1* may inhibit breast alveolar development and epithelial cell differentiation, and lead to breast cancer. However, the accurate mechanisms need further study.

### Glioma

Glioma is the most common and aggressive form of adult primary brain tumors, accounting for 80% of malignant brain tumors [23, 24]. Despite all surgical efforts in combination with chemo and radiotherapy, gliomas are still incurable, and the prognosis remains dismal. Undoubtedly, a deeper understanding of the molecular mechanisms of glioma initiation and progression, identifying novel biomarkers and therapeutic targets, will contribute to develop more effective therapy for glioma.

Gao et al. [25] found *ZFAS1* was upregulated in glioma tissues and cell lines and higher *ZFAS1* expression in glioma tissues was significantly correlated with advanced tumor stage and poor OS. Then, knockdown of *ZFAS1* in glioma cell lines significantly suppressed proliferation, migration and invasion. Lv et al. [26] reported that *ZFAS1* expression was markedly upregulated in glioma tissues and tightly correlated with clinical stage and shorter survival. Then they found knockdown of *ZFAS1* in glioma cell lines could promote apoptosis, and inhibit cell proliferation, migration, and invasion. Furthermore, *ZFAS1* silencing could result in cell cycle arrest at the G0/G1 phase in glioma cell lines. Taken together, these data showed *ZFAS1* might act as a valuable prognostic biomarker and potential therapeutic target for glioma.

### Colorectal cancer

According to the latest statistics, colorectal cancer (CRC) is the third most commonly diagnosed cancer in the United States [27] and the fifth most diagnosed cancer in China [1, 34]. Although the 5-year survival has improved in the rich countries during the past decades, it remained less than 50% in the most developing countries, including China [28, 29]. Therefore, identifying novel functional biomarkers for the early diagnosis in CRC patients are important for improved its survival.

Thorenoor et al. [30] reported *ZFAS1* was significantly up-regulated in CRC tissues compared to paired normal colorectal tissues. Then, *ZFAS1* silencing decreases proliferation and tumorigenicity of CRC cell lines through inducing G1-arrest of cell cycle. Wang et al. [31] found *ZFAS1* expression was significantly upregulated in CRC tissues compared with adjacent noncancerous tissues, and was higher in metastatic tumor tissues than in corresponding primary CRC tumor tissues. In addition, they found higher *ZFAS1* expression in CRC was positively correlated with lymphatic invasion, TNM stage and poorer prognosis. Moreover, they found knockdown of *ZFAS1* inhibited the metastasis of CRC cell lines and mouse models of metastasis. Xie et al. [32] reported that *ZFAS1* expression was significantly upregulated in CRC tissues and cell lines, higher *ZFAS1* expression was significantly associated with Helicobacter pylori infection, lymph nodes metastasis, advanced TNM stage and poor overall survival of CRC patients. Then they found *ZFAS1* inhibition could markedly suppress CRC cell lines proliferation and invasion both in cell lines and mouse models of proliferation and metastasis. Another study by Fang et al. [33] found *ZFAS1* was up-regulated in colonic cancer tissues compared with adjacent mucosa, and its expression level was significantly correlated with TNM stage, vascular invasion, and lymph node metastasis. In addition, they found knockdown of *ZFAS1* impeded proliferation, invasion, and promoted apoptosis of colonic cancer cell lines. The above data showed *ZFAS1* might act as a valuable prognostic biomarker and potential therapeutic target for CRC.

### Gastric cancer

Gastric cancer (GC) is one of the most common digestive malignant tumors and ranks as the second cause of death by malignancy worldwide [34, 35]. Although the progressive diagnosed methods and standard chemotherapy protocols have been established, prognosis of GC patients is still very poor [36]. Thus, there is still a pressing need to elucidate the molecular mechanisms underlying gastric cancer progression, as well as to identify key biomarkers and develop effective targeted therapies.

Zhou et al. [37] reported *ZFAS1* was up-regulated in both tissues and plasmas of GC patients, as well as GC cell lines compared with paired normal tissues, plasmas or normal gastric cell lines. Another study by Nie et al. [38] reported that *ZFAS1* was overexpressed in GC tissues, and its increased level is associated with shorter survival. In addition, knockdown of *ZFAS1* impaired proliferation and induced apoptosis of GC cell lines, and inhibited the tumorigenicity of GC cells in mice. Pan et al. [39] found *ZFAS1* expression was elevated in tumor tissues, serum and serum exosomes of GC patients and GC cell lines. Then, the increased *ZFAS1* expression was significantly correlated with lymphatic metastasis and TNM stage. *ZFAS1* knockdown inhibited the proliferation and migration of GC cell lines. On the contrary, *ZFAS1* overexpression promoted the proliferation and migration of GC cells. Moreover, they found *ZFAS1* was present in exosomes and could be transmitted by exosomes to enhance proliferation and migration of GC cell lines. These data indicated *ZFAS1* may be involved in regulation of EMT in GC progression, *ZFAS1* might serve as a potential biomarker and/or therapeutic target for GC.

### Hepatocellular carcinoma

Hepatocellular carcinoma (HCC) is one of the most prevalent and aggressive malignancies worldwide [35], with incidence rates continuing to increase rapidly [2]. In many cases, HCC is diagnosed at an advanced stage, which limits treatment options and affects prognosis [40]. So it is urgent to seeking new effective biomarkers for early diagnosis and effective therapy of HCC.

Li et al. [41] reported that *ZFAS1* was frequently amplified in HCC, and associated with intrahepatic and extrahepatic metastasis and poor prognosis of HCC. Another study by Wang et al. [42] found *ZFAS1* was markedly downregulated in HCC tissues and cell lines and overexpression of *ZFAS1* inhibited the proliferation and induced the cell apoptosis in HCC cell lines. These data suggested *ZFAS1* may be as a new prognostic biomarker and target for clinical management of HCC.

### Ovarian cancer

Ovarian cancer is one of the most common and lethal gynecological malignancies [43]. Despite the advances in the treatment such as surgical technique, chemotherapy regimens and radiotherapy, the 5-year survival rate of ovarian cancer is still not satisfied [44]. So, it is imperative to elucidate the molecular mechanism of this disease and identify the biomarkers for treatment of ovarian cancer.

Xia et al. [45] reported *ZFAS1* was upregulated in epithelial ovarian cancer tissues, and higher expression of *ZFAS1* was correlated to poorer prognosis of patients with epithelial ovarian cancer. Then, they found *ZFAS1* play a critical role in promoting the proliferation, migration, and chemoresistance of epithelial ovarian cancer cell lines. They further found *ZFAS1* promoted proliferation, migration, and chemoresistance of epithelial ovarian cancer. Liu et al. [46] found higher expression of *ZFAS1* was significantly associated with insensitivity of platinum-based chemotherapy by the analysis of The Cancer Genome Atlas (TCGA) and Gene Expression Omnibus (GEO) datasets. Furthermore, they found the *ZFAS1* expression was upregulated in cisplatin treated ovarian cell lines. These findings suggested that *ZFAS1* serve as novel markers, therapeutic targets and participating in platinum resistance in ovarian cancer.

### Other cancers

In recent days, some studies also reported that ZFAS1 plays a vital role in other cancers such as melanoma, non-small cell lung cancer (NSCLC), osteosarcoma (OS), esophageal squamous cell carcinoma (ESCC), acute myeloid leukemia (AML) and natural killer/T cell lymphoma (NKTCL).

Wei et al. [47] reported that the expression of *ZFAS1* was significantly increased in melanoma tissues compared to adjacent non-cancerous tissues. They found the higher *ZFAS1* expression were correlated with tumor thickness, lymph node metastasis, tumor stages in poor disease-free survival time (DFS) or over survival (OS) of melanoma patients. Then, knockdown of *ZFAS1* by siRNAs suppressed melanoma cell line proliferation. Tian et al. [48] reported *ZFAS1* was upregulated in NSCLC tissues, and higher expression in more advanced tumor tissues. Then, they found higher *ZFAS1* expression were significantly associated with advanced tumor differentiation grade, lymph node metastasis, advanced TMN stage and a poorer prognosis. Liu et al. [49] found the expression of *ZFAS1* was significantly overexpressed in OS tissues and cell lines, and upregulation of *ZFAS1* was significantly associated with unfavorable prognosis of OS patients. They further found that *ZFAS1* enhanced the growth and metastatic ability of OS cell lines and growth and metastatic model of mice. Shi et al. [50] reported *ZFAS1* expression was significantly upregulated in ESCC tissues compared with the corresponding adjacent normal tissues. ESCC patients with higher *ZFAS1* expression had a advanced histological grade, T stage and a poor prognosis. In addition, they developed a nomogram integrated clinicopathological factors and *ZFAS1*, could accurately predicted the prognosis of lymph node-negative ESCC patients without preoperative chemoradiotherapy. Guo et al. [51] reported *ZFAS1* was upregulated in AML cell lines compared with T lymphocytic leukemia cell line or Burkitt’s lymphoma cell line, then they found knockdown of *ZFAS1* impeded proliferation, and promoted apoptosis of AML cell line. Baytak et al. [52] found *ZFAS1* was overexpressed in NKTCL tissues and *ZFAS1*-associated genes are enriched in pathways regulating proliferation and survival. The above data showed ZFAS1 might act as a valuable prognostic biomarker and potential therapeutic target for Melanoma, NSCLC, OS, ESCC, AML and NKTCL.

## Regulatory mechanisms of ZFAS1 in human cancers

**Fig. 1:**
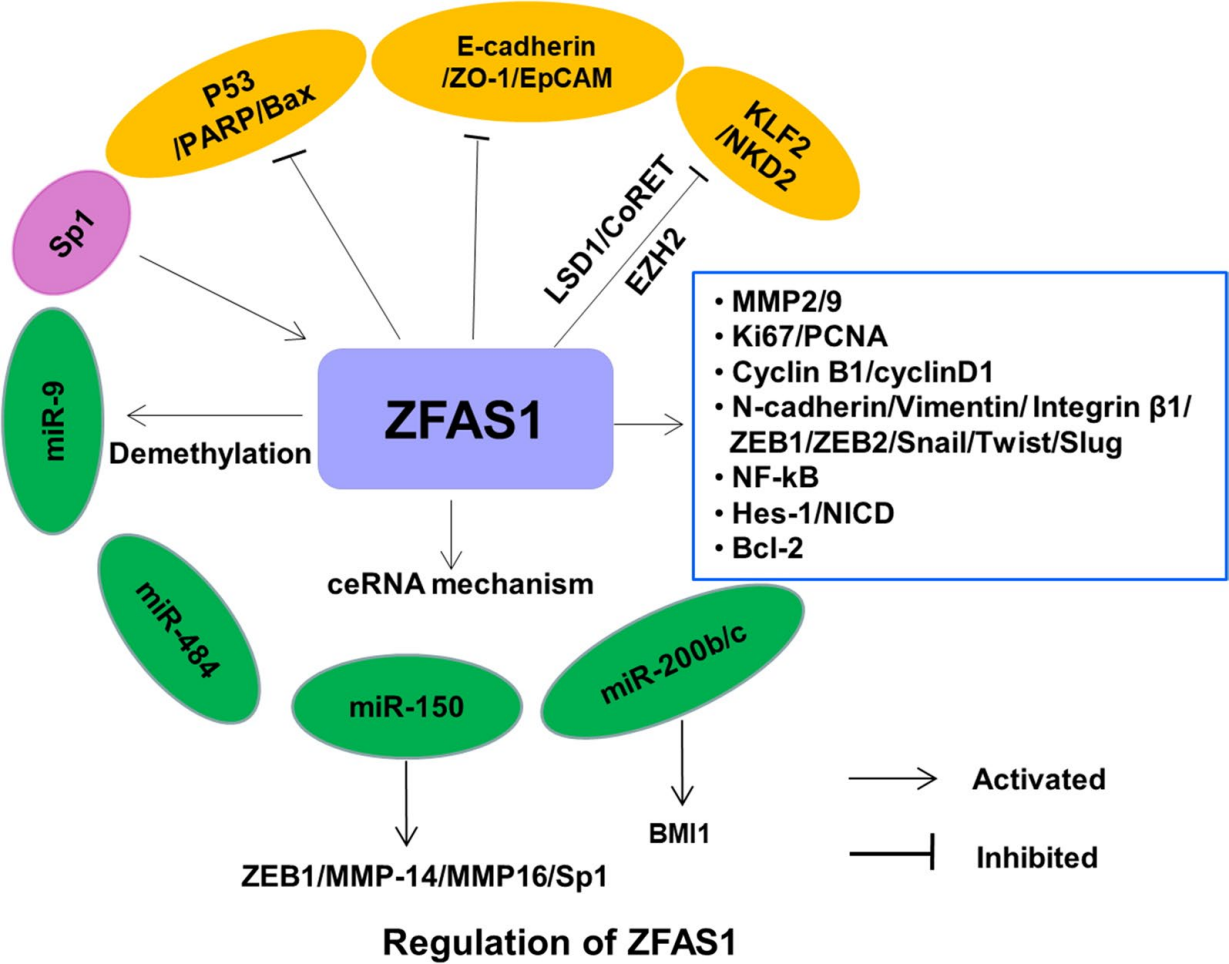
The regulatory mechanisms of ZFAS1 in human cancers

Overexpression of ZFAS1 in various cancers has been shown to exert oncogenic functions through regulating cancer-related phenotypes, such as promoting cell proliferation, migration, invasion, inhibiting apoptosis in different cancer types (Table 1). The mechanism by which ZFAS1 mediates such actions is complex and involves multiple factors (Fig. 1).

### Promotion of epithelial–mesenchymal transition (EMT)

As we know, EMT is deemed to be the essential process of cancer progression, enhancing tumor migration and invasion [53–55]. During the EMT process, epithelial cells lose epithelial status, apicobasal polarity and cell–cell adhesion so as to transform into mesenchymal cells [56, 57]. Positive regulation of EMT, manifested as down-regulation of epithelial markers (e.g. E-cadherin) and up-regulation of mesenchymal markers (e.g. vimentin), by knockdown of ZFAS1 has been demonstrated in glioma, colorectal cancer and gastric cancer. These data indicate *ZFAS1* may be key activator of EMT process. However, how *ZFAS1* regulate the notch signaling, direct or indirect, is unknown until now.

### Activation of notch signaling pathway

The functions of notch signaling are commonly played in cooperation with other pathways during tumorigenesis. Previous study has showed that notch signaling is associated with EMT for cancer progression [53]. Gao et al. [25] found knockdown of *ZFAS1* affected the expression of the notch signal-related proteins including HES family bHLH transcription factor 1 (HES-1) and notch intracellular domain (NICD), which indicate *ZFAS1* may be another key activator of notch signaling. However, how *ZFAS1* activates the notch signaling, direct or indirect, is unknown until now.

### Regulation of proliferation, invasion and metastasis related genes

Just as we know, proliferation-related gene such as Ki67, PCNA, Cyclin B1, CyclinD1, PARP, Bcl2 and Bax, invasion and metastasis related genes such as MMP2, MMP9 and MMP14 play the key role in tumorgenesis and progression. It has been demonstrated that *ZFAS1* could regulate the expression of above gene, thus promote the tumorgenesis and progression of various cancers such as melanoma, glioma, colorectal cancer and gastric cancer [26, 30, 37, 47]. However, whether these effect are direct or indirect, is unknown until now.

### Acting as competing endogenous RNA (ceRNA)

Competing endogenous RNA (ceRNA) is a novel regulatory mechanism whereby lncRNAs function as ceRNA to sponge microRNAs (miRNAs) and regulate their downstream signaling pathways has been proposed and confirmed preliminarily [58, 59]. It was demonstrated that ZFAS1 exerted as ceRNA to enhance the expression of proliferation, invasion and metastasis related genes, such as ZEB1, MMP-14, MMP16, BMI1, Sp1 and ZEB2 by competitively sponging miR-150, miR-200b or miR-200c [41, 49], which indicated ZFAS1 became a new lncRNA functioning as ceRNA in cancer.

### Other mechanisms

Wang et al. [42] found miR-9 was lowly expressed in HCC tissues and positively correlated with *ZFAS1* expression. Meanwhile, significant downregulation of the methylation of CpG island in miR-9 promoter and upregulation of miR-9 expression were observed when *ZFAS1* was overexpressed in HCC cell lines. Nie et al. [38] found that ZFAS1 could simultaneously interact with enhancer of zeste homolog 2 (EZH2) and lysine specific demethylase (LSD1)/REST corepressor-1 (CoREST) to repress the transcription of kruppel-like factor2 (KLF2) and naked cuticle 2 (NKD2) transcription by RNA immunoprecipitation and RNA pull-down experiment in GC cell line. Moreover, Liu et al. [49] found that SP1 functions as an upstream activated factor of ZFAS1 in osteosarcoma.

## Conclusions and future perspectives

Since its discovery, *ZFAS1* have been widely investigated in various human malignancies. *ZFAS1* is upregulated and plays an oncogenic role in most types of tumors. However, *ZFAS1* is downregulated in breast cancer, and may function as a tumor suppressor. Such discrepancy might be caused by distinct gene expression backgrounds in different tumors. In addition, the function of *ZFAS1* in HCC is controversial, which needing further study. The mechanisms by which *ZFAS1* mediates its actions is complex and involves multiple factors, including sponge action of miRNA, the effect on EMT, and the effect on cell cycle and apoptosis (Fig. 1). However, relatively little is known about the mechanism mediating upregulation of *ZFAS1* in human tumors. A better understanding of its upstream regulation and downstream signaling could therefore provide insights to devise better strategies to target *ZFAS1*. The influences of genetic, epigenetic and environmental factors on *ZFAS1* also warrant further investigation.

Pertinent to clinical practice, *ZFAS1* might serve as an independent prognostic marker whose upregulation foreshadows a poor clinical outcome. Nevertheless, almost all studies did not include an independent cohort for validation. The prognostic and diagnostic performances of *ZFAS1* against existing markers in different ethnic groups have also not yet been systemically assessed. Furthermore, the chemical stability of *ZFAS1* in biological samples (e.g., serum) is also unclear. In future studies, the diagnostic and prognostic performance of *ZFAS1* should be evaluated head-to-head with existing clinicopathological and serological markers in larger cohorts in order to accelerate its clinical utilization. Moreover, identification of small-molecule inhibitors of *ZFAS1* through chemical library screening is also necessary to translate findings from basic science into clinical benefits.
